# An uncharted territory of sexualized drug use: exploring the dynamics of chemsex among young and adolescent MSM including self-identified gay men in urban Dhaka, Bangladesh

**DOI:** 10.3389/fpsyg.2023.1124971

**Published:** 2023-06-22

**Authors:** Samira Dishti Irfan, Golam Sarwar, Junyed Emran, Sharful Islam Khan

**Affiliations:** Program for HIV and AIDS, Infectious Diseases Division, International Centre for Diarrhoeal Diseases Research, Bangladesh (icddr,b), Dhaka, Bangladesh

**Keywords:** chemsex, drug use, SRHR, adolescent, youth, gay

## Abstract

Global and local literature depicted the pervasiveness of chemsex among men who have sex with men (MSM), yet there is limited evidence on adolescents and youth. Though literature showed their engagement in chemsex, further exploration is warranted about their socio-sexual contexts and implications. Therefore, this article explored the contexts and implications of chemsex on young and adolescent MSM. This article is extracted from qualitative research evidence, and research data are triangulated by programmatic evidence on adolescent and young MSM from two ongoing pilot interventions. The key motivational factors for engaging in chemsex were primarily rooted in the dynamics of their peer networks. Specifically, the onset of drug use is predominantly attributed to curiosity toward experimentation with methamphetamine, peer influence, propensity to lose weight, and increasing courage to approach potential sexual partners. Moreover, they continued taking drugs as it enhanced their sexual performance, thus perpetuating chemsex. Additionally, the findings revealed several sexual implications of methamphetamine, e.g., bolstering their sexual “stamina,” increasing their propensity toward sexual violence, and reducing their decision-making abilities and judgment, thus collectively decreasing condom use. In essence, chemsex is considerably driven by their socio-sexual contexts, thus perpetuating sexual risk behaviors and compromising sexual health outcomes. Therefore, harm reduction interventions targeted need to be designed keeping in mind their socio-sexual dynamics and age.

## 1. Introduction

Sexualized drug use (popularly known as chemsex) has become an increasingly pervasive drug-taking practice. As defined by the literature, chemsex is “the use of specific drugs before or during planned sex to facilitate, initiate, prolong, sustain and intensify the encounter” (Maxwell et al., 2019, p. 1). However, chemsex is a dynamic phenomenon which could vary geographically, culturally, and temporally (Maxwell et al., 2019). Although harm reduction and other reports have revealed that chemsex has increased over the last decade (Harm Reduction International, 2021) and has become the most widely used drug in Asia and the Pacific (APCOM, 2013), it remained a lingering phenomenon that has even dated back to the 1990s, when gamma hydroxybutyrate (GHB) became popular in gay clubs in Western settings because of their ability to bolster sexual pleasure. Throughout the 21st century, the prominence of methamphetamine increased among the gay community, particularly MSM. Although this term was never used, it was a phenomenon that was eventually coined as chemsex for the first time since 2010 (Stuart, 2019). After the first chemsex parties occurred in 2011 in London, United Kingdom, the term gradually gained widespread popularity (Hakim, 2019).

The prevalence of chemsex differs depending on the subgroup and also varies based on the heterogeneity in definitions assigned by different scholars (Tomkins et al., 2019). Some of the most popularly used drugs in chemsex include methamphetamine, mephredone, and gamma hydroxybutyrate/butyrolactone (GHB/GBL; Bourne et al., 2014; Harm Reduction International, 2021). However, in the context of Asia and Pacific, some additional drugs have been classified under the chemsex umbrella because of their potential to initiate and enhance sexual encounters. For example, the Asia Chemsex Platform, a regional coalition that was established in 2020, has classified alkyl nitrates (i.e., poppers) as drugs used in chemsex, along with other drugs such as ecstasy, ketamines, etc. These drugs originate from various groups which produce a different set of physiological effects. For example, methamphetamines, ecstasy, and mephredone produce belong to the group of amphetamine-type stimulants which typically activate the sympathetic nervous system, thus inflicting sleeplessness, loss of appetite and increased sexual performance (APCOM, 2013). The onset of the effect of methamphetamines start within approximately 15 min of intranasal intake/inhalation (Cruickshank and Dyer, 2009) and “high” lasts for 8–12 h (Meredith et al., 2005). Whereas the desired effects of mephredone starts within 15–45 min after ingestion with an oral dose of ~200 mg, and the effect lasts for approximately 2–3 h (Papaseit et al., 2016). On the other hand, GHB/GBL are inhibitors which are known to inhibit ejaculation within a short period of time and sexually charge people (Sex Health Wellbeing, 2021). Following intake, performance and behavioral effects of GHB initiates approximately after 10–30 min. and can last from 1–6 h based on the dose. The duration of effect can be influenced by simultaneous use of other drugs (Giorgetti et al., 2017). However, the co-ingestion of ethanol and GHB may causes an adverse interaction between these two sedative drugs (Busardo and Jones, 2015). On the other hand, poppers, Alkyl nitrites with an onset within a few seconds and effect lasts for few minutes, are used as sex aids, invoke a feeling of “high,” and reduce the pain associated with anal intercourse (Choi et al., 2005; Demant and Oviedo-Trespalacios, 2019).

Chemsex is a socially constructed concept, which is contingent on the popularity and availability of specific drugs, as well as the users’ preferences (Maxwell et al., 2019). The literature has also reflected that behavioral patterns and practices of chemsex are divergent based on varying cultural contexts and socio-demographic characteristics of the users, which include sexual orientation and age.

In particular, the existing evidence base indicates that males having sex with males (MSM) are more likely to engage in chemsex compared to their heterosexual counterparts (Maxwell et al., 2019). Studies from several countries showed that a proportion of MSM, ranging from 17% to 38.9%, practice chemsex, especially the use of amphetamine-type stimulants such as methamphetamine (Maxwell et al., 2019; Sousa et al., 2020). Particularly in Asia, the prevalence of these practices among MSM ranges from 3.1% to 30.8% (Nevendroff et al., 2021). However, there are globally acknowledged concerns about the interconnectedness between chemsex and high-risk sexual behaviors which could exacerbate the HIV and STI transmission potential of these populations by perpetuating unprotected anal intercourse, increasing the length and frequency of sexual intercourse and increasing the number of sexual partners (Kenyon et al., 2018; Pakianathan et al., 2018; Pufall et al., 2018; UNODC, 2018). In addition, a study conducted on MSM in Spain revealed that engagement in chemsex considerably compromises the health-related Quality of Life of MSM (Ruiz-Robledillo et al., 2021). Likewise, Tomkins et al. (2019) indicated growing evidence of the association between chemsex and mental health concerns among MSM, thus indicating the potential biopsychosocial risk factors for MSM who are involved in chemsex. A systematic review reported that chemsex is associated with anxiety, depression and psychosomatic symptoms (Tomkins et al., 2019). All of these complexities are tied to the broader umbrella of sexual and reproductive health and rights (SRHR), therefore from the SRHR perspective, it is important to ensure that MSM-focused interventions account for issues related to chemsex.

Chemsex is also receiving increasing international research and programmatic attention, containing “an emerging body of knowledge on different aspects of the behavior” (Maxwell et al., 2019, p. 2), which also includes age. There is a growing body of literature exploring the chemsex situation among young and adolescent MSM, albeit to a limited extent compared to their adult counterparts. For example, the P18 cohort study based in the United States evinced significant associations (AOR = 4.98) between sexualized drug use and unprotected anal intercourse (Ristuccia et al., 2018). Similarly, latent class analysis has revealed that adolescents were more vulnerable to sexualized drug use as they are more likely to be exposed to chemsex at a young age (Tan et al., 2021a,b). Chemsex is likely to precipitate a steep increase in HIV prevalence among young MSM in some countries in Asia such as Indonesia and Malaysia (The Global Fund, 2020).

In Bangladesh, there is limited research about the chemsex situation among MSM and other gender and sexually diverse populations. A recent qualitative study conducted in Dhaka, Bangladesh among MSM and transgender women (locally known as *hijra*) revealed that the participants would often engage in chemsex for various reasons, primarily enhancing their sexual performance, intensifying their sexual pleasure, augmenting their capacity to uptake more clients to generate more income, and increasing their sexual pride (Khan et al., 2019). However, the same study also revealed that chemsex could perpetuate behaviors that could be detrimental to their SRHR-related outcomes, such as increasing coercive sex, enhancing group and serial sex, engaging in transactional sex with unknown clients, multiple sex partners, and condomless anal intercourse which increased their vulnerability to sexually transmitted infections (STIs; Khan et al., 2019, 2022).

However, further research about the risky sexual behaviors of MSM is warranted in a setting which criminalizes male-to-male sex and any other sex acts besides heteronormative sex, as per the legal framework of Bangladesh (GOB, 1972). Because of the criminalization of male-to-male sex, MSM often remain hidden and hard-to-reach out of the fear of identity disclosure and potential legal repercussions. These barriers could contribute to their ability to access and utilize safer sex practices, yet there is scant knowledge about this phenomenon among this group. Therefore, the sexual risk behaviors of MSM need to be further explored within their criminalized context. Another layer of vulnerability has been added in the context of the criminalization of drug use. In particular, according to the Narcotic Control Act of 2018, where the possession of at least 200 g of methamphetamine could lead to life imprisonment (GOB, 2018), thus exacerbating their vulnerability to partake in safer drug-using behaviors.

There is currently a paucity of global and regional evidence that explores the underlying contexts and implications of sexualized drug use among young and adolescent MSM including self-identified gay, particularly in an emerging generation that presents new sets of SRHR vulnerabilities and healthcare access challenges (Halkitis and Singer, 2018). Similarly, the only available evidence in Bangladesh explores the reasons and effects of methamphetamine among older MSM, thus the terrain of youth and adolescent MSM including gay men remained unexplored. There is a current paucity of evidence about the contexts and implications of chemsex among this group in Bangladesh and many regional countries around the globe. However, as indicated by the existing global evidence, these populations may present their own set of complexities, unmet needs, and risky sexual behaviors (Tan et al., 2021a,b). Additionally, the literature has highlighted the importance of collecting in-depth empirical evidence for obtaining a nuanced and authentic understanding of the co-occurrence of drug use and sexual activity (Weinhardt and Carey, 2000; Vosburgh et al., 2012). This will guide researchers in identifying priority areas which need to be intervened in existing and future SRHR interventions. Therefore, this article aims to explore the contexts and implications of sexualized drug use (i.e., chemsex) among young and adolescent MSM and self-identified gay men.

## 2. Materials and methods

### 2.1. Premises of the qualitative evidence

The findings of this article are extracted from a qualitative study that was conducted in urban Dhaka, Bangladesh to explore the patterns and key driving factors for chemsex, particularly methamphetamine use, as well as the implications of chemsex on the sexual lives of MSM and *hijra* (Khan et al., 2019, 2022). That qualitative study consisted of 30 in-depth interviews (IDIs) and six focus group discussions (FGDs) with the target population (described below), and 15 key-informant interviews (KIIs) with HIV intervention service providers, harm reduction specialists, and academicians. These interviews were conducted under the framework of phenomenology, as it is a useful theoretical tool for conducting an in-depth reflective inquiry to derive nuanced meaning from the participants’ experiences (Chamberlain, 2009; Neubauer et al., 2019) regarding the contexts and complexities of chemsex.

The evidence presented in the previous two articles (Khan et al., 2019, 2022) prepared from the aforementioned qualitative study, focused on MSM aged 25 years and above who practiced chemsex, and belonged to a compromised economic background. Thus, there remains a paucity of information from adolescents and young adults (i.e., aged between 18 and 24 years) MSM and transgender women, receiving HIV services from the drop-in centers (DICs), operated by the NGOs. Moreover, we did not have data on the self-identified gay men who belong to the urban socioeconomic background, are relatively educated, hard to reach, and generally do not seek services from the DICs due to the contexts described in the background section of this article. To foster a deeper understanding of the complexities of chemsex among the young and adolescent MSM, we revisited the data from the IDIs and FGDs of the qualitative study. We analyzed the data from seven IDIs and two FGDs specifically conducted among young and adolescent MSM of the qualitative study described above.

### 2.2. Application of programmatic evidence in this article

While analyzing the data generated from the qualitative research study (described above), some additional information was perceived necessary for encapsulating a comprehensive picture of the chemsex scenario among the young and adolescent group. To ensure this, we collated evidence from an ongoing pilot chemsex intervention that provided harm reduction services including counseling to MSM including self-identified gay men. This created opportunities for counselors and service providers to learn about their reported chemsex-related concerns which may have transcended the nature of our research data but elicited an authentic and enriched understanding of the complexities of chemsex. We had another intervention that was operated within the framework of a virtual outreach program for providing HIV prevention services to self-identified gay men. Thus, we also gathered information on chemsex from gay men who reported this while taking services through the virtual platform. In this context, we leveraged the opportunity to complement the qualitative research findings with program and service-related information through counselors and service providers from both interventions. This was done through triangulation, which is considered an integral tool for strengthening the scientific rigor of qualitative evidence (Mertens and Hesse-Biber, 2012; Heale and Forbes, 2013; Patton, 2014).

The triangulation approach used in this article includes data source, methodological, investigator and analytical triangulation (Heale and Forbes, 2013; Patton, 2014; Noble and Heale, 2019). Specifically, the research data have been collected by sociologists, anthropologists, demographers, and public health specialists, whereas programmatic information from the participants has primarily been exchanged with counselors (who are clinical psychologists), physicians, and service providers from the target group, all of who were adequately sensitized and attuned to the complexities and sensitivities of this community. This approach was posited to enhance the authenticity and trustworthiness of the data (Mertens and Hesse-Biber, 2012). We attempted to reflect the diversity produced by this triangulation approach in the results section.

### 2.3. Participant and settings

Both the qualitative study and pilot programmatic interventions were conducted in urban Dhaka, Bangladesh among gender and sexually diverse people (i.e., MSM and transgender women) although the evidence presented in this article is specifically focused on the young and adolescent MSM. These young and adolescent MSM emerge from two main socio-demographic groups. One group emerges from lower socioeconomic groups and often partake in transactional sex in order to generate income. They often uptake services from the NGO-operated HIV prevention interventions. On the other hand, there is a group of young and adolescent men who self-identify as gay men. They typically emerge from higher socioeconomic backgrounds and reside in affluent neighborhood. Due to the fear of identity disclosure and their reluctance to associate with the former group, they do not visit the HIV prevention service centers.

The research and programmatic data were both collected from a total of four NGO-operated HIV prevention intervention service delivery points called Drop-in Centers (DICs) which render HIV and STI prevention and management services for gender and sexually diverse people. These DICs have also recently been providing two pilot interventions (described above). These DICs are located in the north, south, west and eastern parts of urban Dhaka city. Although this paper has presented two different distinct groups of populations, there are no overlaps between these groups.

### 2.4. Data collection techniques and approaches

The evidence presented in this paper is based on extensive literature review on chemsex-related issues, along with a variety of data collection techniques which are explained in the Table 1. From the qualitative study, data were extracted from two FGDs and seven IDIs from young and adolescent MSM aged between 18-24 years old, including self-identified gay men. The depth generated from the IDIs and the overall societal insights elicited from the FGDs complemented each other to provide a comprehensive picture of the chemsex scenario.

**Table 1:** Data collection methods, investigators and participant categories.

SL	Data source	Data collection technique	Participant type(s)	Data collector(s)	Rationale
1	Research	In-depth interview (IDI)	Young and adolescent MSM and self-identified gay men	Anthropologists, sociologist, public health experts	To elicit an in-depth understanding of the underlying contexts, socio-sexual dynamics and effects of drug intake on their sexual activities.
2		Focus group discussions (FGDs)			To provide a breadth of understanding of the normative and societal perspectives regarding chemsex among this group.
3	Pilot interventions (same strategies were used for both of the interventions)	Informal discussions	Young and adolescent MSM and self-identified gay men	Psychologists, clinical psychologists, and data collector from the target population	To explore and understand the socio-sexual and cultural dynamics of chemsex, drug use patterns, sexual behaviors and their service needs
4			Service providers of DICs (i.e., DIC managers, peer educators, medical assistant)	Psychologists, clinical psychologists, counselors, physicians, anthropologists	To understand and explore the reasons and implications for partaking in chemsex on a broader community level
5		Service register	Quantitative information	Counselors and physicians belonging to the pilot project	To understand the number of participants who have engaged in chemsex
6			Field diaries (Qualitative)	Counselors and physicians belonging to the pilot project	To understand the common socio-sexual scenarios leading to chemsex, drug use patterns, and sexual behaviors
7		Programmatic monitoring and evaluation visits	Young and adolescent MSM and self-identified gay men	Physicians, counselors and programmatic experts (e.g., sociologists, anthropologists, demographers, etc.)	To understand the service needs and uptake, as well as the overall dynamics of chemsex

Qualitative information was derived similarly from ongoing monitoring and evaluation, service field diary notes, and day-to-day interactions between the clients and the counselor, from both of the pilot interventions. To assess their service needs and understand the overall social context, we collect information from the participants who belong to the adolescents and young groups through informal discussion. Through these discussions, we were able to understand their socio-cultural dynamics, sexual practices, drug use patterns, and other related issues experienced by these populations. We maintain a service register from which we collect quantitative information of the participants. Moreover, the service providers were expected to maintain service-related field notes which served as another valuable source of programmatic evidence in qualitative textual form. Additionally, during the ongoing monitoring and evaluation component of the intervention, we solicited their experiences and facilitate informal discussions with the participants.

### 2.5. Data analysis

Firstly, the data from the qualitative study were analyzed using the conventions of thematic analysis stipulated by Braun and Clarke (2006). A small subset of transcripts was read, from which an initial set of themes were generated. Thereafter, we searched for relevant themes, reviewed and defined them and then presented these themes as findings (Braun and Clarke, 2006). A collaborative coding framework was applied where the authors jointly decided the thematic categorization of the data and reached a consensus (Saldaña, 2015). Decision-trails were documented accordingly and any disputes in the coding categorizations were decided by the senior author. Secondly, as triangulation entails the use of multiple data collection techniques and analytical approaches (Golafshani, 2003), we also convened the research evidence with the programmatic data and thematically categorized them based on the codes that were generated in the collaborative coding framework.

Moreover, to reflect the degree of triangulation in our analysis, under several themes, we have included participant anecdotes from a diversity of sources. This approach is expected to encapsulate multiple perspectives about chemsex, regardless of the profession of the data collector, or the objective of that specific data collection technique. For example, the same reasoning for chemsex may have been reflected in both an IDI and a counseling session in the chemsex harm reduction intervention even though the former was aimed at collecting research data whereas the latter intended to understand the complexities of a patient from a service provider’s perspective. Qualitative scholars have underpinned data collectors’ tendencies to attach their own interpretations to findings, thus triangulation has been posited as an avenue for minimizing subjective bias.

To ascertain scientific rigor, this triangulation initiative was also complemented by peer debriefing sessions where the service providers who collected the data and the authors exchanged their perspectives regarding the interpretation of the findings (Connelly, 2016).

### 2.6. Ethical considerations

The qualitative study already received ethical approval from the Ethical Review Committee of the Institutional Review Board of the International Centre for Diarrhoeal Diseases Research, Bangladesh before its inception. Moreover, in order to use the information extracted from the participants of these two interventions, we have attained ethical approval from the Ethical approving authority of icddr,b. Moreover, before conducting any counseling session or any informal discussions, we informed the participants that the information they reveal could be used for scientific reporting purposes, and their identities and all information would be kept fully confidential. With their verbal approval, we only collected information that is required for the interventions to be operated successfully. To ensure anonymity and confidentiality, their names were only available to the authorized counselors and physicians who work within the pilot intervention. Before presenting these data to the researchers who analyzed this data, their names were removed, thus adding another layer of informational confidentiality. Moreover, all of the interview and conversational data was de-identified and stored in a password-protected computer.

## 3. Results

The triangulation of the research and pilot programmatic evidence presented a variety of contexts of drug use, and, hence, chemsex among young and adolescent MSM, including self-identified gay men. In the interest of preserving their confidentiality, the participants’ names have been removed.

### 3.1. Use and patterns of drugs in chemsex

Programmatic evidence collected from the quantitative information of the service register from the pilot interventions revealed that a total of 74 participants from both of the interventions combined fall within the age group between 18 and 24 years. In addition to the quantitative information, the participants have also been inquired about their drug use patterns during the counseling sessions. In addition, the IDIs and FGDs have revealed some insights about their drug onset and continuation behaviors and patterns.

According to the quantitative information collected from the service register, there are a total of 74 participants from both of the pilot interventions who are aged between 18 and 24 years old, of whom 42 were found to use drugs at least once in their lifetime. Among the user’s, types of used drugs given in Table 2.

**Table 2:** Types of drugs used by the participants.

Name of the drug	*N* = 42
	*n* (%)
Alcohol	40 (95.2)
Cannabis	25 (59.5)
Methamphetamine	5 (11.9)
Poppers	4 (9.5)
Sedatives	2 (4.8)
Phensedyl	2 (4.8)
Cocaine	0 (0)
Ecstasy	0 (0)
Viagra	0 (0)
Polydrug use	26 (61.9)

The most important drug used (*n* = 5) was methamphetamine in the context of these populations. Although they have used other drugs such as alcohol (*n* = 40) and cannabis (*n* = 25), these have not been classified within our definition of chemsex in this paper. Table 3 represents the type of drugs used in a sexual context.

**Table 3:** Types of sexualized drugs used by participants.

Name of the sexualized drug	*N* = 42
	*n* (%)
Alcohol	25 (89.3)
Cannabis	9 (32.1)
Methamphetamine	4 (14.3)
Poppers	4 (14.3)
Cocaine	0 (0)
Ecstasy	0 (0)
Viagra	0 (0)

Among the 42 participants who reported using any kind of drug, 28 responded that they used those drugs in a sexual context. However, since poppers were found to be taken among our participants (n = 4) for the purpose of activating their sexual experience, they have also been discussed as a chemsex drug in this results section. Table 4 represents drug use characteristics among those who engaged in chemsex.

**Table 4:** Drug use characteristics among chemsex practitioners.

Variables	*N* = 5
	*n* (%)
**Route of administration**	
Inhaled	5 (100.0)
Oral	0 (0)
Sniffed	0 (0)
Injecting	0 (0)
**Frequency of drug use**	
Every day	0 (0)
Every week	1 (20.0)
Every month	1 (20.0)
Every 3 months	2 (40.0)
Every 6 months	1 (20.0)
Every 12 months	0 (0%)
**Number of drugs used**	
Single drug	2 (40.00%)
Poly drug	3 (60.00%)
**Individuals who had negative experiences due to chemsex practice (a loss of consciousness, vomiting, headache, not remembering, non-consensual sex)**	
No	4 (80.00%)
Yes	1 (20.00%)

100% of the participants who practiced chemsex took methamphetamine through inhalation and no one admitted to taking the drug through the mouth, injecting, and sniffing.

Moreover, during the in-depth interviews and focus groups, the participants were asked about their preferred mode of administration of taking methamphetamine. All of the participants who took methamphetamine reported that they preferred inhaling. For example, one of the focus group participants mentioned that, “This (snorting) is how we take the drug. It has always been popular among our group so I do not know any other way.” (Participant Y, 21–22 years old).

This was also corroborated after a reading of the counselors’ field diaries. In addition, an informal discussion conducted with one of the participants, a university student, described how he took Yaba in the following way:

We usually inhale or snort the drug on a piece of paper or old money note. We put the Yaba tablet on the foil and then light it up until it burns. It brings out a flame and then we either inhale or puff it out (snort). We consider it a complete process (Participant M, 20–22 years old).

### 3.2. “*They were trying it out so I feel like I had to as well*”: the inception of their drug-taking journeys in relation to their social circles

Notably, the participants’ onset into drug use were rarely marked by a need to embellish their sexual experience. Rather, their prime motivating factors for drug use were predominantly marked by the influence and dynamics of their peer networks. The programmatic and research findings, alike, depicted their social culture within their community. Many of the participants represented higher socioeconomic backgrounds (i.e., lived in an affluent neighborhood where their parents held white-collar jobs and supported them) where social prestige, urbanization, high educational status and wealth were considered the key staples of social assimilation.

They often congregated in exclusive, invite-only social gatherings where drugs were considered a social adhesive to an enjoyable experience with their peers. On the other hand, however, the MSM and transgender women in the lower socio-economic groups presented their own set of contextual circumstances. For example, their drug using and sexual behaviors were driven by their need to enhance their sexual behaviors either to survive in their transactional sex careers or improve their sexual performance within their intimate partnerships. Moreover, drug use was driven by their need to bolster their sexual pleasure. It is worth noting that neither of these groups used social media to initiate social contact, rather, they would meet through in-person social gatherings or, in the case of the lower socioeconomic MSM, through the service centers or cruising spots where they would commonly congregate.

While some of the participants from both the research and pilot interventions directly initiated their drug-taking journey with methamphetamine (locally known as Yaba), others’ drug onsets were marked by other intermediary drugs such as cannabis, poppers, etc. and then they gradually transitioned into Yaba.

It is worth noting that many of the participants started experimenting with drugs out of curiosity, after witnessing the actions of their peers. While their peers did not directly pressure them on several occasions, the participants nevertheless felt the need to take these drugs after noticing that their socially prestigious peers were taking these drugs. This created a scenario where participants attached social connotations of drugs as a social symbol. After taking the drugs invoked pleasurable feelings associated with intoxication, they spiraled down a pathway of drug dependency. For example, during a counseling session with the clinical psychologist, Participant M relayed his onset of taking Yaba in the following way:

During my rag day at university, I noticed that some of my new friends and seniors were taking Yaba. Some of them are even drug dealers who sell drugs to their university juniors. I was curious about what they were taking so I felt like I had to try it out (the drug) as well. So, this is where my journey of taking drugs began (Participant M, 20–22 years old).

On the other hand, however, Participant H relayed the same motif of peer influence but described a different scenario where his friends casually coaxed him into drug use. When one of the data collectors from the self-identified gay community had a conversation with Participant H during his monitoring visit, he described his drug-using behaviors in the following way:

I noticed that my friends in my residence hall were smoking (inhaling) something. It looked really interesting and I was interested in trying it out. But of course, I was scared. But my friends told me that it feels so good, I should definitely try it out. And this is the beginning of my life with drugs (Participant H, 20–22 years old).

Within the context of their social circles, drug-taking not only served as a gateway to their peer groups, but it was also a perceived social activity that consolidated friendships. Specifically, some drug-taking activities, such as sharing lighters and drug-taking apparatus, symbolized social intimacy and fraternity. In other words, drugs were perceived as the adhesive that cemented their social circle. For example, during an in-depth interview, one of the participants mentioned:

One of the main activities in our group revolves around taking Yaba together. During this time, we also chat, gossip and joke together. Yaba is what has made our group stronger. There is something special about sharing the same lighter and pipe when taking Yaba, it makes our bond even stronger (Participant X, 22–24 years old).

Moreover, our findings from the informal discussions and counseling sessions for both interventions have revealed other social connotations of Yaba use, such as the catalyst for a seemingly attractive appearance. For instance, a few of the participants mentioned that they started taking Yaba as a weight loss aid. Like most other social settings, being thin was perceived as a proponent to social prestige and increased popularity within the peer network. In one particular example revealed in a counseling session for the harm reduction intervention, Participant F followed suit after one of his university roommates started pursuing this practice:

My roommate used to regularly take Yaba. I was always wondering why. He told me Yaba helped him lose weight quickly. Being an overweight person, I always felt self-conscious about how I looked. So that is what drove me to take Yaba. It was a very easy ticket to losing weight. But once I started, I became highly addicted and there was no way for me to stop (Participant F, 21–23 years old).

The findings, from the research interviews and programmatic informal discussions, alike, also revealed a prominent culture of informal drug peddlers who would primarily target newcomers because they believed that their naivety about drugs would easily enable them to sell lower-quality drugs at higher prices. Therefore, this alludes to the social hierarchy and motifs of seniority which interplayed within the social circles of our participants. The participants attached social meaning to these drugs, citing them as a status symbol, thus making their peers more willing to emulate them. As one of the participants mentioned:

Yaba is a symbol of wealth and style among our community. Only the rich and cool kids seem to take it. By taking this drug, you give off the impression of being smart, a risk-taker, modern, classy, adventurous, basically anything you could ever want. That is why this drug seems so appealing (Participant R, 22–24 years old).

### 3.3. Underlying contexts for practicing drug use in sexualized settings (i.e., chemsex)

The findings illuminated various contexts which have ultimately driven them toward sexualized drug use (i.e., chemsex). As described in the previous section, many of them initiated drug use as a product of their social environment, rather than the desire to enhance their sexual lives. However, their continuation of drug intake and regular practice of chemsex were often attributed to their surrounding socio-sexual contexts, which are described in this theme. In particular, this section specifically describes various events, circumstances, and perspectives which lead to chemsex.

#### 3.3.1. “Drugs helped me approach them”: using chemsex as an aid to entice sex partners in parties

The participants from the informal discussions, counseling sessions and research interviews explained that their social lives often revolved around “parties” which constitute limited-guest, invite-only gatherings within tight-knit social groups. These parties are often situated in secluded venues such as participants’ residences, hotel rooms, etc. to circumvent identity disclosure as a gay man, as this identity is criminalized and stigmatized within the context of Bangladesh. Although these parties commence with music, food, and socializing, as the parties progress through the night, alcohol and drug use predominate in the party setting.

According to the majority of the participants, drugs were perceived as a precursor to a conducive environment for sexual activity. Most of the participants noted initially feeling shy and apprehensive about socializing and acquainting themselves with potential sex partners, despite reporting feeling attracted to them. Therefore, upon the suggestion of their peers and witnessing them engaging in similar acts, they resorted to taking Yaba to bolster their courage to approach their peers. From the perspective of the participants who regularly attended these parties, their social status and reputation are dependent on whether they can successfully participate in sexual activities and “score” a sexual partner. Therefore, under these circumstances, they often gravitated toward drug use. As one of the participants mentioned during a counseling session in the harm reduction intervention for chemsex:

I often feel shy to approach and talk to other guys in the party even though some of them are really hot. So, I need something to help me get the courage to approach them. Because of Yaba, not only can I approach them, but I can also talk freely with them all night. This drug made me talk non-stop. But of course, one thing leads to another, and then we ended up f***ing each other. Only Yaba was able to make this possible (Participant T, 18–20 years old).

This was also corroborated by another informal discussion that was conducted during a monitoring visit for the virtual outreach intervention.

My sister’s guy friends would often have get-togethers, and I found them quite hot. Although I cross-dress sometimes, I felt shy about letting myself loose in front of them. I could not even entertain them properly in my sister’s house when my sister was not around. But Yaba helped break that barrier for me. After I took my first whiff of Yaba with them, I was finally able to make out with one of the guys (Participant S, 19–21 years old).

According to the research interviews, similar scenarios resonated among the young and adolescent MSM who originated from lower socioeconomic backgrounds as they wanted to confidently approach clients for sex work in order to garner additional income.

Similarly, an academician, who has extensive experience in the chemsex discipline among MSM and other gender sexually diverse people, corroborated that stimulant drugs, such as Yaba, has the potential to increase one’s audacity and talkativeness. However, he also highlighted that this practice carries overall risks in terms of affecting their sexual health outcomes, as it increases their inclination toward unsafe sex. As he mentioned:

Yaba increases their ability to hold a conversation and makes them more willing to socialize. Therefore, they are able to easily tackle social settings under the influence of drugs. However, these often turn into sexual encounters, which could be risky for them, as drugs affect their judgment and decision-making abilities (Academician working with drugs and sexual minorities).

#### 3.3.2. A means to forget the agony associated being gender and sexually diverse

Some participants in both the research interviews and counseling sessions stated that they started using a sexualized drug to deal with social stress associated with being sexually minor people. Many participants reported feelings of low self-confidence and esteem due to being marginalized and ostracized from their families on the grounds of mannerisms which are typically manifested by gay men of that community. As a result, they often felt alienated, thus they reported self-medicating with drugs such as Yaba.

In addition to coping with social stress, methamphetamine also helped participants increase their sexual self-confidence. Before initiating sexualized drugs, a participant reported being unable to confidently communicate and negotiate with sexual partners. He stated that:

Before sex work I take Yaba (methamphetamine). Without Yaba, I feel like I cannot talk and attract partners. Earlier, I did not have the confidence to talk to a partner for sex. I used to worry that my partner would say bad things to me or beat me up. But after taking Yaba, I can approach any man and talk about sex without hesitation (Participant R, 22–24 years old).

#### 3.3.3. “Now I can smoothly have sex”: using chemsex to facilitate painless anal intercourse and sexual satisfaction

Many of the research and pilot intervention participants reported that it has been difficult to practice anal intercourse for a long duration because of the pain resulting from the friction, especially if they do not have lubrication on hand. Therefore, upon inspiration from their peers, they opted for taking drugs, primarily Yaba, to lessen the pain often felt during anal intercourse. As a result, this enhanced their ability to practice prolonged intercourse, thus ultimately enhancing their sexual performance. Their increased performance instilled a sense of pride, knowing that they were able to achieve a sexual conquest within their social setting, thus elevating their social status within their circle. During one of the IDIs, a participant described his predicament in the following way:

Anal sex used to be so painful before, it felt dry and every time I would go back and forth in the butt, it would just hurt. This is where Yaba has felt like a lifesaver. Now I can smoothly have sex. Also, now I feel even more satisfied because I can have sex for a long time (Participant M, 20–22 years).

Likewise, a participant who had an informal discussion with a data collector from his community relayed the same sentiment in the following way:

I feel like drugs helped me achieve something. During these parties, I could not have sex for a long time before but now this has changed. I was able to make another man satisfied, so I felt like I won (Participant K, 22–24 years old).

At the same time, they revealed that they experienced unprecedented degrees of sexual satisfaction when having sex under the influence of drugs. They did not derive the same magnitude of sexual pleasure and magnitude when having sex normally. Some participants struggled to describe their feelings and experiences with methamphetamine, whereas others briefly described their sexual experiences, feelings and sensations. For instance, when inquired about their experience with a sexualized drug, one participant from a focus group spoke of distinct sensations, emphasizing the motifs of warmth, heat, and lust, “My whole body gets hot with lust after taking Yaba. Suddenly I feel like I am the king of sex (*kamer raja*). It is really difficult to explain in words” (Participant K, 22–24 years old).

Some of the participants also shared that other chemsex drugs, especially poppers, helped them to experience wild pleasurable sex which would not have been otherwise attainable sans the influence of drugs.

These scenarios were also substantiated by a service provider in an existing SRHR intervention for MSM who relayed that the participants of their intervention would take Yaba due to similar circumstances. Specifically, they raised concerns about MSM feeling anal pain, therefore they perceived Yaba as a viable antidote, particularly for bolstering their sexual performance.

#### 3.3.4. “I feel a lot closer to my sex partners”: an aid for augmenting intimacy in intercourse

Not only were drugs reported as a pathway for alleviating the social and physical barriers of anal intercourse, but they were also perceived as an aid for increasing the degree of sexual and emotional intimacy within their intercourse act. Before they regularly took drugs, the research and programmatic participants, alike, opined that it was difficult for them to achieve emotional intimacy with their sex partners, particularly if they were new, unfamiliar partners. Most of the participants reported that drugs amplified the degree of pleasure that they felt during anal intercourse. Because of this, they were able to practice prolonged anal intercourse, thus incurring a sense of emotional satisfaction from the ability to satisfy their partner. On most occasions, this has perpetuated their dependency toward drugs. As one of the participants originating from a lower socioeconomic class mentioned during a counseling session with the clinical psychologist:

Before (taking drugs regularly), I had a hard time enjoying sex, especially with partners I don’t really know. But now that I am taking Yaba and other drugs, I feel a lot closer to my sex partner. Now I can have sex for a long time and feel a lot more pleasure than before (Participant K, 22–24 years old).

Moreover, findings from both the conversational data and the counselors’ field diaries revealed that these heightened feelings of sexual pleasure perpetuated a cycle of dependency where they could no longer derive pleasure from sexual intercourse without drugs. Therefore, they felt compelled to continue taking drugs. This sentiment was echoed by one of the participants in the following way:

I keep on wanting more of this drug. Once I started taking the drug, I understood why it was so special (*eitar moja ta bujhe gesi*) so now I cannot have any fun during sex without this drug. I have now become addicted (Participant S, 19–21 years old)!

Participants also reported that sexualized drugs would stall the ejaculation that they would have achieved if they engaged in foreplay. However, some other participants reported that they can no longer have sex without sexualized drugs which are illustrated in the following IDI anecdote:

Me and my sexual partner have been having sex on Yaba for a long time. Now it’s a situation where my partner can only have sex with me if he takes Yaba, otherwise, he cannot have sex with me anymore (Participant K, 18–20 years old).

### 3.4. Implications of chemsex in the sexual setting

The findings also revealed that chemsex has modified their sexual behaviors, particularly in socio-sexual settings. On several occasions, drug use engendered their risky sexual behaviors, particularly because these sexual encounters did not involve condom use. These practices have the potential to elevate their likelihood of transmitting HIV and STIs, thus exacerbating their overall sexual health outcomes. This section specifically highlights the ways in which chemsex has shaped their sexual experiences and practices, primarily in relation to their social setting and the dynamics of their peer networks.

#### 3.4.1. “I have been watching a lot of hot stuff on porn and wanted to try it out”: increase in desire toward sexual experimentation

The counselors field diaries have revealed that chemsex is perceived as an avenue for fulfilling the participants’ erotic fantasies, whether with known or unknown partners. This was also corroborated by the research interviews, counseling sessions and other informal discussions. Oftentimes, the participant reported the desire to enact the activities they have watched on pornographic media which often fall within the Bondage and Discipline, Dominance and Submission, Sadism and Masochism (BDSM) category. However, on most occasions, they reported feeling shy or apprehensive about this practice while sober, especially with unfamiliar partners. On the other hand, chemsex was perceived to alleviate that barrier. Based on the anecdotes of one of the participants from the counseling session:

I have been watching a lot of hot stuff on porn where people are spanking each other, tying each other up, etc. I really want to try that stuff out but sometimes I feel too shy to do these things. But Yaba changes me completely. I no longer feel that barrier. I tried it out with a few partners and we had a really good time (Participant K, 18–20 years old)!

This specific population represents a sexual dynamic where male-to-male sexual partnerships consist of a “top” (i.e., insertive) and “bottom” (i.e., receptive) in the relationship. Conventionally in this population group, the top and bottom partners remain fixed in their roles. However, chemsex has increased the fluidity in their sexual roles. According to the participants, this is attributed to their increased willingness to sexually experiment. As one of the participants mentioned during his conversation with the data collector from the self-identified gay community:

I have always played the top role with my man. But now that I take drugs regularly, I find it too boring. I really want to try something new. I have always wondered what it is like to be f***ed from behind. So, this is where I started playing the bottom role (Participant I, 20–22 years old).

In addition, some of the participants explained that chemsex heightened their propensity to practice group sex, which was deemed as a relatively rare occurrence while sober. One of the participants in an informal discussion mentioned that “Once we take drugs, we lose our minds and before you know it, three or four of us are f***ing together” (Participant I, 20–22 years old). This situation particularly resonated with parties which involved chemsex.

Some anecdotes, from both the field diaries and conversational data, alike, also revealed the recurring incident of golden showers where two sexual partners often go to the bathroom after completing their sex act. In this instance, one partner often micturates over another, and thus he considers it as a shower. In a more atypical case, a client hired a male sex worker and brought him to his residence. Thereafter, he took Yaba. Initially, he performed sex with his wife in front of the hired male sex worker, followed by a sexual encounter with the hired man.

#### 3.4.2. “I ignored his cries and kept on going”: increase in ferocity during sexual encounters

The participants described that the intake of Yaba, poppers, and other drugs have altered their state of mind to the extent that they were more likely to become violent in their sexual activities. From the perspective of the participants, chemsex has transformed them into a “ferocious animal” (Participant Z, 20–22 years old). This sub-section uncovers the motif of sexual violence in two dimensions: in terms of making them more likely to non-consensually approach a new partner; and practicing rough or coercive sex with an existing partner.

As mentioned before, these social circles often constitute a seniority dynamic where the younger MSM are more likely to appease their seniors. Under the influence of drugs, senior members of the group often leverage that dynamic to approach their juniors for sex, sometimes to the extent of blackmailing or threatening them if their demands are not fulfilled. These incidents rarely involved condom use. As one of the IDI participants who were victimized by this practice mentioned:

They (the seniors) go mad after taking drugs. The other day, one of them approached me for having sex. I was not interested at the time, so I refused. But he would not accept no for an answer. So, he threatened me that he would tell my whole family I was gay if I did not listen to him. He seemed so angry at the time I simply could not say no. So he f***ed me (Participant S, 19–21 years old).

Moreover, the interview findings, as well as the field diary notes, revealed that chemsex made some of the participants sexually violent to the extent that they made rough or coercive sex a part of their existing sexual partnerships. Even if the partner was experiencing anal pain or anal bleeding from the prolonged sexual activity, the participants reported that they could not stop, as their sexual pleasure superseded every other circumstance. In one of the anecdotes, a participant mentioned:

He (my sexual partner) was bleeding from behind from all the rough sex, but it was impossible for me to stop even though he was crying in pain. Because of this drug, I just wanted to keep on going on and on the whole night. So, I ignored his cries and just kept on going (Participant I, 20–22 years old).

#### 3.4.3. “I’ll go mad unless I find someone to f***”: chemsex posing a risk of reducing judgment and decision-making abilities

The majority of participants who have taken drugs before a sexual encounter reported that chemsex altered their state of mind to the extent that they were unable to make informed decisions. Rather, the consumption of Yaba and other similar drugs have intensified their sexual cravings where they immediately needed to satisfy their sexual desires at that moment. This situation was particularly applicable to parties where some participants intended to achieve at least one sexual conquest, otherwise, it would affect their reputation within their respective social circles. As one of the IDI participants mentioned:

This drug makes me sex crazy. I cannot think about anything else except sex. At that moment, I feel like I will go mad unless I find someone to f***. And what is even worse is that I am at this party where people are f***ing each other left and right. I need to find someone too or else I’ll feel left out. I do not want to be the only one without a sex partner (Participant K, 18–20 years old).

It is of mention that the participants’ decision-making abilities were clouded to the extent that condoms remained a secondary concern. In the context of the aforementioned parties where sex has remained a primary staple, at that moment, they prioritized fulfilling their sexual urges. As one of the participants expressed during a counseling session with the clinical psychologist:

Who cares about condoms? At that time, the only thing that mattered to me is fulfilling that burning sexual craving that is eating me up inside. The drugs have me so crazy for sex that I do not even think about condoms. I know I should be wearing them, but what can I do (Participant Z, 20–22 years old)?

On rare occasions, participants who took drugs for a longer time (i.e., more than 3 years) reported feeling anal pain from prolonged sex. Therefore, this further fuelled their reluctance to use condoms because they were more willing to continue sex as opposed to safeguarding their sexual protection. In addition, some of the participants who have been taking Yaba for a long time perceived that Yaba was necessary for facilitating an erect penis, to the extent where their penis even failed to erect after taking multiple tablets of Yaba.

## 4. Discussion

This is one of the few qualitative articles of its kind that explores the socio-sexual contexts and implications of chemsex on adolescents and young MSM and self-identified gay men whose primary social circles lie within this community. It is worth noting that these findings consist of two denominations of youth and adolescents. One group originates from the lower socioeconomic strata whose sexual behaviors are often driven by the need to bolster their sexual performance to generate income. Whereas, another group comprises an affluent social cluster of self-identified gay men who embody a different drug-taking and sex culture.

These findings primarily presented a contextualized scenario of the social attributes, nature of the sexual partnerships and social settings which have perpetuated chemsex. On the other hand, the majority of the literature has merely illuminated the relationship between drug use and risky sexual behaviors, often without exploring these crucial social contexts. Yet, in order to intervene in the sexual harms associated with chemsex, it is crucial to also understand the nuanced complexities of their socio-sexual contexts, which is also important for promoting better SRHR outcomes. Hence, these findings have bridged a pressing knowledge gap whereby we have begun to understand the circumstances shaping their sexual behaviors, especially among young and adolescent self-identified gay men who remain relatively less explored as a population group in Bangladesh and other settings. It is worth noting that their elusive nature is primarily attributed to the criminalization of both male-to-male sex and drug use (explained in the introduction section) which has collectively fuelled their self-stigma. Therefore, this paper highlights some contexts of drug use that are tied to the stigma associated with their criminalized status, a phenomenon that is yet to be underpinned in other literature.

One of the strengths of this qualitative research was its application of various types (i.e., methodological, data source, and investigator) triangulation, particularly by complementing the research with the programmatic data from the pilot interventions. This approach has allowed for ensuring the validity and trustworthiness of data collected from multiple data sources and investigators. Even though the research and programmatic initiatives were conducted with a different set of objectives, where the former was aimed at understanding a scenario and the latter was intended to understand the patients’ needs and complexities, they produced similar data even from data collectors with differing professions and, hence, different sets of subjective biases.

Another unique facet of this article was its ability to unravel another culture that previously did not receive adequate research attention. For the self-identified gay group, these contexts are predominantly rooted in the culture constructed by their peer networks. Moreover, the findings have delved into how chemsex has shaped their sexual lives, in the context of their social and sexual settings. These findings have been thematically categorized into various dimensions of chemsex. While the first part describes their socio-sexual contexts, the results section then segues into an analysis of how chemsex has perpetuated various risky sexual behaviors. In Bangladesh and its surrounding region, the literature has predominantly focused on the culture of older MSM, who have their own predefined set of cultural dynamics and typically originated from lower socioeconomic backgrounds (Chakrapani et al., 2019; Khan et al., 2019, 2021; Bhambhani et al., 2021). Specifically, in partnerships, this group of MSM typically embodies traditionally masculine and feminine roles, where the latter typically wears feminine clothing, displays effeminate mannerisms, etc. On the other hand, the same masculine and feminine dispositions do not exist among the young and adolescent population groups explored in this article. Rather, their sexual dynamics are reminiscent of MSM population groups in other countries in the Asia-Pacific region and worldwide.

In particular, a previous qualitative study conducted by Khan et al. (2019, 2021) revealed some underlying reasons for methamphetamine use among masculine and feminine MSM from older groups. While that study uncovered the motifs of enhancing sexual performance, prolonging intercourse and decreasing the friction associated with intercourse, these sexual performance-related issues did not resonate to the same extent among our participants as they were younger and more able. Moreover, Khan et al. (2019) have described that masculine MSM wanted to showcase their masculinity by demonstrating that they can perform sex with multiple partners for prolonged periods of time. Conversely, feminine MSM derived pride in their femininity from being able to entertain multiple consecutive partners. However, these gender roles did not exist among our participants, as there was no traditional masculine and feminine dichotomy.

Moreover, the MSM groups from lower socioeconomic backgrounds often engaged in transactional sex, as one of their driving factors was their need to survive. Therefore, they considered methamphetamine as an aid to uptake more clients. However, this scenario does not resonate with our participants as they do not present the same needs. Rather, since their drug intake is not tied to financial need, the participants perceived drugs as a symbol of wealth and social prestige.

In addition, our findings illuminated a seniority dynamic within the social circle, which drove newcomers into taking drugs to appease their seniors. As a result, our findings illuminated Yaba and other chemsex drugs as an adhesive for their social networks. This particular insight was yet to be highlighted in any other regional or global literature, except for an online ethnography based in Thailand which uncovered a similar dynamic in their “ice parties” (Guadamuz and Boonmongkon, 2018). Although the pink carpet Y cohort study based in Singapore posited that an earlier exposure age to sexual networks may perpetuate chemsex (Tan et al., 2021a,b), they did not elaborate on the interplaying socio-sexual dynamics. Thus, these findings were able to bridge that knowledge gap. Moreover, the Thailand-based study, the pink carpet Y cohort study, and other studies based beyond Bangladesh and its surrounding region have not used social media to initiate contact within their socio-sexual networks.

Nevertheless, some of our findings about the contexts of chemsex are aligned with the extant regional and global literature. For example, the research conducted so far on youth and older MSM, alike, substantiated that participants engage in chemsex to enhance their performance in the sexual setting (Halkitis et al., 2005; Nakamura et al., 2009; Maxwell et al., 2019; Tan et al., 2021a,b). Moreover, studies in other countries such as the United Kingdom, Canada, and Thailand depicted the relationship between social influence from virtual and in-person communities and the onset of sexualized drug use (Wei et al., 2012; Patten et al., 2020). These studies have also identified the motifs of social capital and affiliation with sexual networks (Wei et al., 2012; Tan et al., 2021a,b), which have been explored in further detail in our assessment, thus reflecting this dynamic among youth for the first time. However, other studies merely pinpointed this relationship whereas our findings described the socio-sexual contexts surrounding chemsex.

Additionally, our participants’ lived experiences regarding the implications of chemsex have also been found in other literature on older MSM. Since the existing literature on young and adolescent MSM is relatively limited, there is a paucity of data on how drugs have shaped their sexual lives. However, the literature based in Bangladesh and other countries revealed that methamphetamine and other drugs have influenced their sexual lives by increasing their degree of sexual violence, decreasing their likelihood of using condoms, and increasing their propensity to practice transactional sex (Guadamuz and Boonmongkon, 2018; Jalil et al., 2022; Khan et al., 2022). However, while studies that are focused on youth have highlighted that youth are more likely to gravitate toward social experimentation, the previous research did not highlight the increased degree of sexual violence that resulted from drug use. Moreover, the extant literature did not delve into issues such as coercive sex, BDSM, sexual exploitation at parties, etc.

### 4.1. Implications of the findings, recommendations, limitations and future direction

The findings of this qualitative exploration have illustrated various scenarios of chemsex that have emerged from the socio-sexual dynamics among young and adolescent MSM. However, many of these contexts and situations have perpetuated sexual risk behaviors such as engaging with multiple sex partners, practicing rough and experimental sex and forced non-consensual sex. As none of these encounters were found to be protected, this could elevate their vulnerability to adverse SRHR outcomes such as HIV, STIs, etc. These risky behaviors could potentially undermine the benefits of the existing interventions for these population groups, particularly the pilot chemsex intervention, in terms of safeguarding their overall SRHR health.

Therefore, the socio-sexual complexities surrounding chemsex need to be intervened in the extant harm reduction interventions. Specifically, since these populations are younger, they are more likely to be influenced by their social circles (Wei et al., 2012). Thus, it would be more effective to embed these complexities within counseling sessions. Moreover, evidence-based interventions could be applied for tackling the harms of chemsex among young and adolescent MSM. For instance, the literature highlighted the promising outcomes of group therapy interventions for promoting safe behaviors among young groups whose lives are closely tied to their social circles (Vaughn and Howard, 2004; Waldron and Kaminer, 2004; Kaminer et al., 2005). This could be because recent research has highlighted that social ties could promote a sense of accountability and positively influence peer behaviors (Tan et al., 2021a,b). Some recommended harm reduction and therapeutic measures include psychoeducational group sessions, skill development training, support groups, and group cognitive behavioral therapy (Substance Abuse Treatment: Group Therapy, 2005).

One of the limitations of this article is rooted in the qualitative methods because of the lack of scope for generalizability of the findings. Rather, because of the qualitative nature of the findings, the evidence is highly contextualized within the study and programmatic settings.

Even though this research has been limited to exploring the implications of chemsex on condom use, there are other paradigms of sexual health beyond the use of condoms. For example, pre-exposure prophylaxis (PrEP) is a scientifically proven biomedical approach for averting new HIV infections. Moreover, research has underscored the benefits of integrating chemsex harm reduction services and PrEP (Maxwell et al., 2022) in order to strengthen the HIV prevention response among this vulnerable population group. Likewise, U = U (undetectable = untransmissible) has been coined as an effective prevention strategy for populations living with HIV (Bernays et al., 2021), therefore this needs to be considered in interventions catered toward these populations considering their risky sexual behaviors by ensuring adherence to anti-retroviral (ARV) medications.

Another avenue for future research could include the assessment of chemsex situation among a larger group, development of an evidence-based and context-specific chemsex intervention in Bangladesh, and piloted under the implementation research to be adapted to the local context. This research approach could help elicit the key implementation opportunities and challenges in this setting, thus guiding the further redesign and refinement of the intervention. The literature has revealed the long-term implications of chemsex which could undermine their physical, mental and/or sexual health, such as anxiety, insomnia, disturbances in mood, audio-visual hallucinations and sexual dysfunction (Rusyniak, 2013; Akindipe et al., 2014; Dolatshahi et al., 2016). If the complexities of chemsex are not addressed in the existing sexual health intervention, chemsex could have the potential to exacerbate their physical and mental health outcomes. This could also serve as another way forward for future research.

## 5. Conclusion

The findings of this qualitative assessment have explored the diverse socio-sexual contexts perpetuating chemsex among young and adolescent MSM and the implications on their sexual behaviors and practices. In essence, chemsex is considerably driven by the social dynamics that interplay within their peer networks. This substantially differs from the older MSM communities in Bangladesh where their propensity to practice chemsex were primarily driven by their need to increase their sexual performance and even generate income to safeguard their livelihood. Therefore, these findings have the potential to contribute insights which could be utilized to construct tailored, context-specific interventions which particularly cater to the unique complexities of these population groups. If such complexities are addressed, this has the potential to improve their sexual health and, thus, overall SRHR outcomes.

## Data availability statement

The raw data supporting the conclusions of this article will be made available by the authors, without undue reservation.

## Ethics statement

The studies involving human participants were reviewed and approved by Ethical Review Committee, Institutional Review Board, International Centre for Diarrhoeal Diseases Research, Bangladesh. The patients/participants provided their written informed consent to participate in this study.

## Author contributions

SI conducted the literature review, drafted and edited the manuscript, conducted the analysis, and facilitated the data collection. GS also conducted the literature review and contributed to the drafting and reviewing of the manuscript. JE facilitated the data collection and analysis and reviewed the manuscript. SK conceptualized the projects, extensively drafted and reviewed the manuscript, and managed the data collection and analysis. All authors contributed to the article and approved the submitted version.

## Funding

This article is based on a research study funded by Stanford University and two pilot interventions funded by The Global Fund Against AIDS, Tuberculosis and Malaria. The literature review for this article was conducted with the support of Department of Foreign Affairs, Trade and Development (DFATD), through Advancing Sexual and Reproductive Health and Rights (AdSEARCH), Grant number: SGDE-EDRMS-#9926532, Purchase Order 742885, Project P007358.

## Conflict of interest

The authors declare that the research was conducted in the absence of any commercial or financial relationships that could be construed as a potential conflict of interest.

## Publisher’s note

All claims expressed in this article are solely those of the authors and do not necessarily represent those of their affiliated organizations, or those of the publisher, the editors and the reviewers. Any product that may be evaluated in this article, or claim that may be made by its manufacturer, is not guaranteed or endorsed by the publisher.
